# Randomized controlled trial of toremifene 120 mg compared with exemestane 25 mg after prior treatment with a non-steroidal aromatase inhibitor in postmenopausal women with hormone receptor-positive metastatic breast cancer

**DOI:** 10.1186/1471-2407-13-239

**Published:** 2013-05-16

**Authors:** Yutaka Yamamoto, Takashi Ishikawa, Yasuo Hozumi, Masahiko Ikeda, Hiroji Iwata, Hiroko Yamashita, Tatsuya Toyama, Takashi Chishima, Shigehira Saji, Mutsuko Yamamoto-Ibusuki, Hirotaka Iwase

**Affiliations:** 1 Department of Molecular-Targeting Therapy for Breast Cancer, Kumamoto University, Kumamoto, Japan; 2 Department of Surgery, Yokohama City University Medical Center, Yokohama, Japan; 3 Department of Breast Oncology, Jichi Medical University Hospital, Shimotuke, Japan; 4 Department of Breast and Thyroid Surgery, Fukuyama City Hospital, Hiroshima, Japan; 5 Department of Breast Oncology, Aichi Cancer Center Hospital, Aichi, Japan; 6 Department of Breast and Endocrine Surgery, Hokkaido University Hospital, Sapporo, Japan; 7 Department of Breast and Endocrine Surgery, Nagoya City University, Nagoya, Japan; 8 Department of Breast Surgery, Yokohama Rosai Hospital, Yokohama, Japan; 9 Department of Target Therapy Oncology, Kyoto University Graduate School of Medicine, Kyoto, Japan; 10 Department of Breast and Endocrine Surgery, Kumamoto University, Honjo 1-1-1, Chuo-ku, Kumamoto 860-8556, Japan

**Keywords:** Refractory to aromatase inhibitor, Toremifene, Exemestane, Breast cancer

## Abstract

**Background:**

After the failure of a non-steroidal aromatase inhibitor (nsAI) for postmenopausal patients with metastatic breast cancer (mBC), it is unclear which of various kinds of endocrine therapy is the most appropriate. A randomized controlled trial was performed to compare the efficacy and safety of daily toremifene 120 mg (TOR120), a selective estrogen receptor modulator, and exemestane 25 mg (EXE), a steroidal aromatase inhibitor. The primary end point was the clinical benefit rate (CBR). The secondary end points were objective response rate (ORR), progression-free survival (PFS), overall survival (OS) and toxicity.

**Methods:**

Initially, a total of 91 women was registered in the study and randomly assigned to either TOR120 (n = 46) or EXE (n = 45) from October 2008 to November 2011. Three of the 46 patients in the TOR120 arm were not received treatment, 2 patients having withdrawn from the trial by their preference and one having been dropped due to administration of another SERM.

**Results:**

When analyzed after a median observation period of 16.9 months, the intention-to-treat analysis showed that there were no statistical difference between TOR120 (N = 46) and EXE (n = 45) in terms of CBR (41.3% vs. 26.7%; *P* = 0.14), ORR (10.8% vs. 2.2%; *P* = 0.083), and OS (Hazard ratio, 0.60; *P* = 0.22). The PFS of TOR120 was longer than that of EXE, the difference being statistically significant (Hazard ratio, 0.61, *P* = 0.045). The results in treatment-received cohort (N = 88) were similar to those in ITT cohort. Both treatments were well-tolerated with no severe adverse events, although the treatment of 3 of 43 women administered TOR120 was stopped after a few days because of nausea, general fatigue, hot flush and night sweating.

**Conclusions:**

TOR120, as a subsequent endocrine therapy for mBC patients who failed non-steroidal AI treatment, could potentially be more beneficial than EXE.

**Trial registration number:**

UMIN000001841

## Background

The goal of treatment for metastatic breast cancer (mBC) is to maintain the quality of life (QOL) and prolong survival of patients. When patients have non-life-threatening metastases that are suspected to be hormone sensitive (i.e., in breast cancer that is estrogen receptor [ER]- or progesterone receptor [PgR]-positive), it is desirable to continue endocrine therapy as long as possible, since the therapy itself has a minimal negative effect on the QOL [1]. Non-steroidal aromatase inhibitors (nsAIs), such as anastrozole and letrozole, have been mainly employed as early recurrent treatment for postmenopausal breast cancer [2,3]. When nsAI treatment fails, it is unclear which endocrine therapy is the most appropriate. Options include selective estrogen receptor modulators (SERMs), fulvestrant, a selective ER down regulator (SERD), and exemestane.

Exemestane (EXE) is a steroidal AI (sAI) with modest androgenic activity, which was studied in a phase II trial after documented progression during treatment with an nsAI, and showed a clinical benefit rate (CBR) of 20-40% [4]. Toremifene (TOR) is a SERM with a reported efficacy for treatment of postmenopausal breast cancer similar to that of tamoxifen (TAM) [5]. The usual dose of TOR is 40 mg given orally once a day, however, high-dose TOR (120 mg a day; TOR120) has been approved for use in Japan. High-dose TOR has been reported to compete with estrogen at the ligand-binding site of the ER, to suppress insulin-like growth factor-1-dependent growth [6] and to have non-ER-dependent anti-tumor effects such as suppression of angiogenesis [7]. In our previous retrospective study (Hi-FAIR study), TOR120 showed a CBR of 45% and ORR of 10% after prior AI [8].

In the present study, we conducted an open labeled, randomized controlled trial for patients with postmenopausal mBC that had progressed following the administration of an nsAI. The effectiveness and safety of TOR120 were compared to EXE.

## Methods

### Study design

The high-dose toremifene (Fareston®) for patients with non-steroidal aromatase inhibitor-resistant tumor compared to exemestane (Hi-FAIR ex) study group consists of experts in breast cancer endocrine therapy from 15 facilities (registry number UMIN000001841). This is a randomized, open labeled trial designed to compare the efficacy and tolerability of toremifene 120 mg to exemestane in postmenopausal women with hormone receptor positive mBC with disease progression after prior nsAI treatment. Study treatment continued until disease progression, intolerable toxicity, or patient decision. Moreover, this trial has a crossover design: if a patient fails one treatment arm, she is switched to the other arm if possible. This data will be analyzed after 12 more months’ follow-up.

The primary end point of the study was clinical benefit rate (CBR). Secondary end points included objective response rate (ORR), progression free survival (PFS), overall survival (OS), and tolerability. The trial was designed to detect superiority of TOR120 compared with EXE in terms of CBR. In the literature, the CBR of TOR120 could be considered about 45% and that of EXE as 30% [8-10]. To prove a probability of 90% that TOR120 was superior 15% superior to EXE, 41 patients were required for each group. To account for dropouts and protocol violations, we planned to recruit 90 patients (45 in each treatment group). Additionally, this trial is thought to be not actually a Phase II trial, but a rather small Phase III trial designed to show a big difference between the 2 groups.

The first analysis was scheduled to take place at 13 weeks after the last case was enrolled in the trial. The crossover data would be analyzed at one year after the first analysis.

### Patients

Key inclusion criteria of this study were as follows; the patients are postmenopausal women (over 60 years old, or over 45 years old with amenorrhea over 1 year and follicle stimulating hormone levels within the postmenopausal range), with breast cancer confirmed by pathological diagnosis, who had progressive disease during or after prior non-steroidal AI, who have at least one measurable site or evaluable bone metastasis, who have ER positive and/or PgR positive tumors in the primary or metastatic site, who have anticipated survival of more than 6 months and WHO performance status (PS) 1 or PS2 due only to bone metastasis. This study included patients with bone only (lytic or mixed) metastatic disease by assessing variation of serum tumor markers and bone imaging, or, if possible, measuring the bone lesions with CT or MRI. Up to one prior chemotherapy regimen for the treatment of advanced/recurrent BC was allowed. Use of tamoxifen for adjuvant treatment and for advanced breast cancer was also allowed.

Exclusion criteria included the presence of other active malignancies, pregnancy or lactation, life-threatening metastatic visceral disease, brain or leptomeningeal metastasis, prior exposure to either TOR120 or EXE, extensive radiation or cytotoxic therapy within the last 4 weeks or being judged inappropriate by physicians. All women provided written informed consent before registration in the trial. The study was conducted in accordance with the ethical principles originating in the Declaration of Helsinki and with local Institutional Review Board approval at each participating center.

The ER, PgR, and human epidermal growth factor receptor 2 (HER2) status of each patient was analyzed at each participating facility, if possible. Generally, ER and PgR were measured by immunohistochemistry (IHC), and positive and negative status was judged on the basis of the standard criteria used at each facility, typically with a cut-off level of 1%. HER2 was assayed by IHC and/or FISH and in accordance with ASCO-CAP.

### Endpoints and methods of evaluation

The tumor reduction effect was evaluated every 8 weeks based on Response Evaluation Criteria in Solid Tumors (RECIST) [11]. A complete response (CR) was defined as the complete disappearance of the measurable lesions; a partial response (PR) as a decrease by 30% or more in the sum of the longest diameters (LDs) of measurable lesions; progressive disease (PD) as an increase of 20% or more in the sum of the LDs of measurable lesions; and long lasting stable disease (long SD) as no change in the size of measurable lesions for 24 weeks or longer. The objective response rate (ORR) was defined as the sum of the frequencies of CR and PR and the clinical benefit rate (CBR) as the sum of the frequencies of CR, PR and long SD. Patients with only bone metastasis were included in the progression analysis by measuring changes in serum tumor markers, such as CEA, CA15-3. Specifically, reduction in tumor markers and complete calcification, with improvement of bone symptoms were judged to be PR.

Adverse events were evaluated using the National Cancer Institute Common Toxicity Criteria, Version 4. Efficacy was judged by the clinicians at each facility. The cases that were thought to be difficult to evaluate were independently reviewed and judged by the clinical trial office, Kumamoto University.

### Statistical analysis

SAS was used for statistical analyses of the correlation between therapeutic effects and clinicopathological factors. Unpaired groups were compared using an unpaired *t*-test and paired groups were compared using Fisher’s exact test. PFS and OS were analyzed using the Kaplan-Meier method and the results were compared by log-rank test.

## Results

### Baseline characteristics and medical history of patients

**Figure 1:**
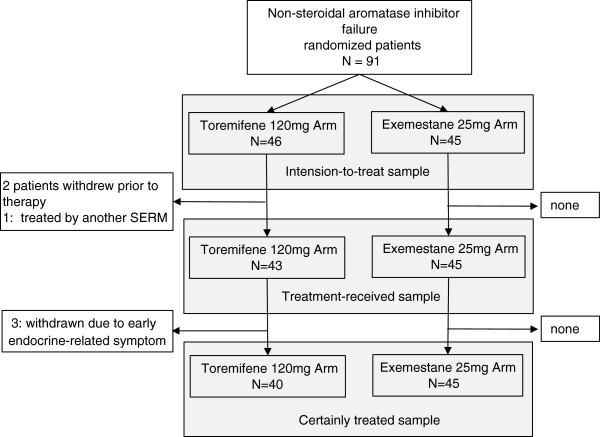
**A consort diagram of this trial.** A total of 91 women was randomly assigned to either TOR120 (n = 46) or EXE (n = 45), who were analyzed as intention-to-treat (ITT) cohort. Three of the 46 patients in the TOR120 were not received treatment, 2 patients having withdrawn from the trial by their preference and one having been dropped due to administration of another SERM. Except for these 3 cases, 43 cases of TOR120 were analyzed the efficacy and safety as ‘treatment-received’ cohort. Three of 43 treatment-received patients with TOR120 were dropped out of TOR120 early because of adverse effects.

Initially, a total of 91 women was registered in this study and randomly assigned to either TOR120 (n = 46) or EXE (n = 45) from October 2008 to November 2011 (Figure 1). These patients were analyzed as intention-to-treat (ITT). Three of the 46 patients in the TOR120 arm were not received treatment, 2 patients having withdrawn from the trial by their preference and one having been dropped due to administration of another SERM. Except for these 3 patients, forty-three patients with TOR120 were analyzed the efficacy and safety as the treatment-received patients (n = 88) (Figure 1).

There was no difference between TOR120 (N = 46) and EXE (N = 45) arm in the patients’ characteristics as listed in Table 1. The median age was 62.2 years (range, 49 to 87) and the median observation period was 72 weeks (range 13–160). Almost of all patients had ER-positive (96%) and HER2-negative (91%) tumors. At the start of the treatment, 20 of the TOR120 arm (44%) and 19 of EXE arm (42%) had visceral metastases, such as metastasis to the lung, liver, or pleura. However, the EXE arm had a slightly greater number of women with bone metastasis (31%, 14/45) than the TOR120 arm (20% 9/46). CBR of the TOR120 arm during treatment with the prior nsAI for ABC was 74% (17/23), and that of EXE arm was 67% (20/30), which was not statistically significant. Approximately 40% of the patients had received chemotherapy for MBC before this trial. Trastuzumab was not used either before or during the trial.

**Table 1:** Patient and tumor characteristics at baseline

**Characteristics**		**TOR120**	**EXE**
Number of the patients		46	45
Age; median (range)		63 (51–87)	62 (49–87)
Follow-up period (weeks); median (range)		69 (13–144)	81 (13–160)
Time elapsed after menopause (years); median (range)		13 (2–37)	13 (1–37)
Body Mass Index; median (range)		22.9 (18.0-35.2)	23.4 (27.7-35.4)
DFI in recurrent cases (months); median (range)		70 (5–188)	60 (1–189)
Estrogen Receptor status	Positive	45	42
	Negative	0	1
	Unknown	1	2
Progesterone Receptor status	Positive	27	31
	Negative	17	11
	Unknown	2	3
HER2 status	Negative	43	40
	Positive	1	1
	Unknown	2	4
Main metastatic lesion			
Visceral disease (main organ)	Lung	10	10
	Liver	7	6
	Pleura	5	3
Non-visceral disease	Bone	9 (20%)	14 (31%)
	Soft tissue	15	12
Performance status (cases)	0,1	45	44
	2	1	1
No. of previous therapies (%)	1	28	29
	2	41	42
	3	24	18
	≥4	9	11
Previous aromatase inhibitor (%)	Anastrozole	48	47
	Letrozole	52	53
Previous treatment with tamoxifen (%)		21	24
Previous chemotherapy (%)		44	38
Sensitivity to previous aromatase inhibitor treatment;			
Clinical Benefit Rate (%)		17/23 (74%)	20/30 (67%)
Duration of previous aromatase inhibitor(months); median (range)		17.1 (2.0-80.8)	17.6 (2.4-65.3)

### Efficacy

**Figure 2:**
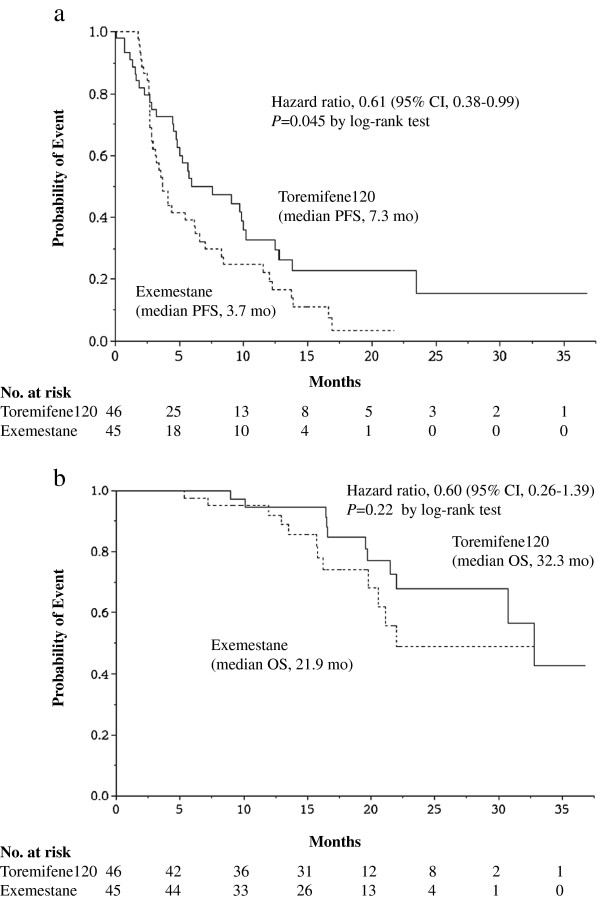
**Kaplan-Meier progression-free survival and overall survival curves. a**. The median progression free survival (PFS) in toremifene 120 mg/day (TOR120) was 7.3 months and that in exemestane 25 mg/day (EXE) was 3.7 months, which showed a statistically significant difference with a hazard ratio of 0.61 (95% Confidence Interval; 0.38-0.99, *P* = 0.045). **b**. Kaplan-Meier overall survival (OS) curves in the TOR120 and EXE. The median OS in TOR120 was 32.3 months and that in EXE was 21.9 months, which showed no statistical difference with a hazard ratio of 0.60 (95% CI; 0.26-1.39, *P* = 0.22) by log-rank test.

Intention-to-treat analysis at a median observation period of 16.9 months showed that there was no statistical significant difference between TOR120 (N = 46) and EXE (N = 45) in terms of CBR (41.3% vs. 26.7%; *P* = 0.14) and ORR (10.8% vs. 2.2%; *P* = 0.083) (Table 2). The median PFS in TOR120 was 7.3 months and that in EXE was 3.7 months, which showed a statistically significant difference with a hazard ratio of 0.61 (95% Confidence Interval, 0.38-0.99), *P* = 0.045 (Figure 2a). The median OS in TOR120 was 32.3 months and that in EXE was 21.9 months, which showed no statistical difference with a hazard ratio of 0.60 (95% CI; 0.26-1.39), *P* = 0.22 (Figure 2b).

**Table 2:** Efficacy analysis

	**Toremifene 120 mg**	**Exemestane 25 mg**	***P *value**
Complete response	1	1	
Partial response	4	0	
Long stable disease (≥ 24 weeks)	14	11	
Stable disease (< 24 weeks)	9	9	
Progressive disease	12	24	
* Withdrew prior to therapy	3*	0	
** Drop out due to early adverse events (not evaluable)	3**	0	
Intention-to-treat cohort	N = 46	N = 45	
Clinical benefit rate% (95% CI)	41.3 (28.3-55.7)	26.7 (16.0-41.0)	0.14
Response rate% (95% CI)	10.8 (4.7-23.0)	2.2 (0.39-11.6)	0.083
Treatment-received cohort	N = 43	N = 45	
Clinical benefit rate% (95% CI)	44.2 (30.4-58.9)	26.7 (13.0-40.1)	0.085
Response rate% (95% CI)	11.6 (5.1-24.5)	2.2 (1.2-16.7)	0.069

*Three patients of all 46 cases withdrew the protocol because of their preference or protocol violation prior to treatment. Except for these 3 cases, 43 cases were assessed as ‘Treatment-received cohort’. **Three other patients dropped out of toremifene 120 mg group due to early adverse events.

CI; confidence interval, ITT; intention-to-treat.

In the treatment-received samples, there was neither between TOR120 (N = 43) and EXE (N = 45) in terms of CBR (44.2% vs. 26.7%; *P* = 0.085) nor ORR (11.6% vs.2.2; *P* = 0.069) (Table 2). Duration of response has not yet been analyzed, because twelve patients (27.9%) of the TOR120 arm and 6 patients (13.3%) of the EXE arm were still being treated at the median observation period of 72 weeks.

### Subgroup analysis

**Figure 3:**
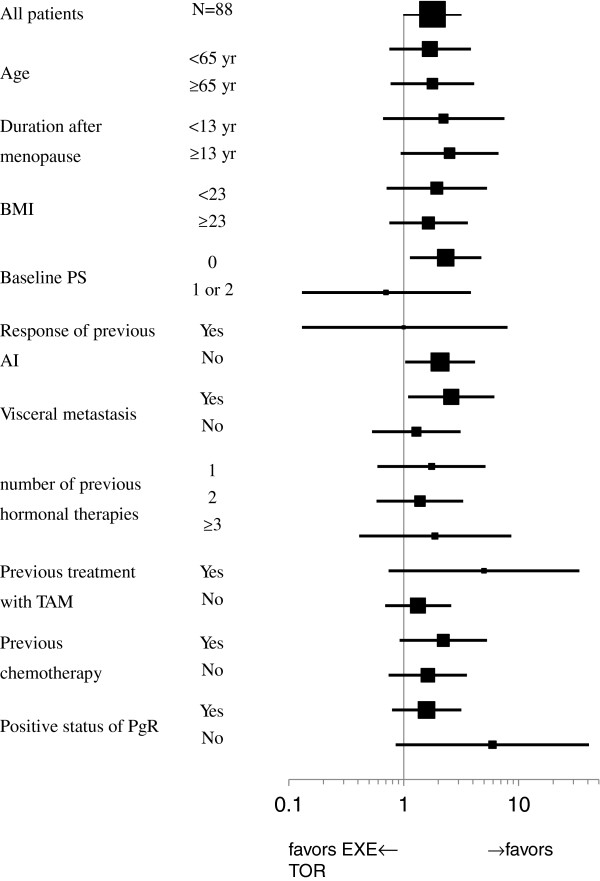
Subgroup analyses of consistent results for clinical benefit; There were no statistical significant differences.

In an investigation of the consistency of treatment effect across the predefined covariates, there were no statistically significant differences. For examples, there was no correlation between the superiority of TOR120 and patients’ age, time since menopause, body mass index, baseline performance status, response to previous AI, presence or absence of viscera metastasis, number of previous hormonal therapies, previous tamoxifen treatment, previous chemotherapy or PgR status (Figure 3).

### Adverse events

Three of 43 women treated by TOR120 withdrew after a few days because of nausea, fatigue, hot flush, and night sweating, which were thought to be endocrine-related symptoms. Except for these 3 cases, both treatments were well tolerated with no severe adverse events (Table 3).

**Table 3:** Adverse Events of toremifene 120 mg and exemestane 25 mg in 88 treated patients

	**TOR120 (n = 43)**		**EXE (n = 45)**	
	**Grade1,2**	**Grade3,4**	**Grade1,2**	**Grade3,4**
Nausea	4*	0	1	0
Fatigue	3*	0	1	0
Hot flush	3*	-	1	-
Night sweating	3*	-	0	-
Vaginal bleeding, discharge	2	-	0	-
Joint pain, disorder	1	-	2	-
Liver dysfunction	1	0	1	0
Exanthema	1	0	0	0

* Three of 43 women treated with toremifene 120 mg were withdrawn from the trial in a few days because of early nausea, fatigue, hot flush and night sweating, which were appeared in same 3 patients.

## Discussion

In our report, a randomized controlled trial was performed to compare the efficacy and safety of daily toremifene 120 mg (TOR120; N = 46) and exemestane 25 mg (EXE; N = 45). Although there were no statistical difference between TOR120 and EXE in terms of CBR (*P* = 0.14), ORR (*P* = 0.083) and OS (*P* = 0.22), the PFS of TOR120 was longer than that of EXE (*P* = 0.045). These results suggested that TOR120, as a subsequent endocrine therapy for mBC who failed non-steroidal AI treatment, could potentially be more beneficial than steroidal AI. Both treatments were well-tolerated with no severe adverse events.

Various endocrine therapies are indicated for postoperative adjuvant therapy of hormone-dependent and recurrent breast cancer [2]. Particularly indicated in cases of postmenopausal breast cancer are treatments that modulate ER function using SERMs and SERDs as well as powerful and highly selective treatments that suppress estrogen synthesis using third-generation AIs. AIs are considered the agent of first choice for endocrine therapy in TAM-failure cases. Especially, nsAIs have been primarily used in postoperative adjuvant therapy or as first line treatment for recurrence. The question arises as to the best choice of subsequent endocrine agent for cases that are refractory to AI.

One option is another AI, such as EXE, which has a steroidal structure and different mechanism of suppressing aromatase activity. Lϕnning et al. [9] reported that the ORR and CBR of EXE after nsAI failure (n = 105) were 4.8% and 20.0%, respectively. In other reports, the CBR of EXE after nsAI failure was around 45% in the second or third line endocrine therapy [1,10]. Chia et al. [12] reported that the ORR and CBR of EXE after nsAI failure (n = 270) as a control arm in their trial were 7.4% and 32.2%, respectively (Table 4).

**Table 4:** The efficacy of subsequent endocrine therapy for advanced breast cancer who have failed to respond to non–steroidal aromatase inhibitor

**Author; Journal, year (Trial name)**	**Line**	**Design**	**n**	**ORR (%)**	**CBR (%)**
Lϕnning; J Clin Oncol, 2000 [9]	2nd ~ 4th	nsAI → EXE	105	4.8	20.0
Iaffaioli; Br J Cancer, 2005 [10]	2nd ~ 3rd	ANA → EXE	50	8.0	44.0
Steele; Breast, 2005 [4]	2nd~ > 4th	nsAI → EXE	114	5.0	46.0
Thürlimann; Eur J Cancer, 2003 [13]	2nd	ANA → TAM	119	10.1	48.7
Chia; J Clin Oncol, 2008 (EFECT) [12]	2nd~ > 4th	nsAI → FUL loading dose	270	7.4	32.2
		nsAI → EXE	270	6.7	31.5
Yamamoto; Breast Cancer, 2010 (Hi-FAIR) [8]	2nd~ > 4th	AIs → TOR120	80	15.0	45.0
Di Leo; J Clin Oncol, 2010 [14]	2nd	AI or SERM → FUL500	362	9.1	45.6
		AI or SERM → FUL250	374	10.2	39.6
Bachelot T: *J Clin Oncol.* 2012 (TAMRAD) [17]	2nd~ > 4th	nsAI → TAM	57	13.0	42.1
		nsAI → TAM + RAD001	54	14.0	61.1
Baselga; N Engl J Med, 2012 (Bolero2) [16]	2nd ~ 4th	nsAI → EXE	239	0.4	18.0
		nsAI → EXE + RAD001	484	9.5	33.4

nsAI: non-steroidal aromatase inhibitor, EXE: exemestane, ANA: anastrozle, TAM: tamoxifen, FUL: fulvestrant, SERM: selective estrogen receptor modulator.

SERMs provide a second option: their various ligand-dependent effects are enhanced by the lower estrogen concentrations in breast cancer tissues that follow treatment with AIs. The ORR and CBR of TAM treatment after nsAI failure (N = 95) were 7.4% (7 cases) and 56.8% (54 cases), respectively [13]. We previously analyzed the efficacy of TOR120 in 80 AI-failure cases: the ORR and CBR were 15% and 45%, respectively, and the median TTF was 7.8 months, which demonstrated satisfactory efficacy outcomes, although this study was retrospective [8] (Table 4). High-dose TOR was reported to compete with estrogen at the site of the ER, to suppress insulin-like growth factor-I-dependent growth [6] and to have non-ER-dependent anti-tumor effects such as suppression of angiogenesis [7].

A third option is the use of much stronger endocrine therapy, such as SERDs, especially high-dose fulvestrant (500 mg on the first day, day 14, and day 28, followed by 500 mg/4 weeks thereafter), which produced a significant increase in PFS compared with the conventional 250 mg regimen [14]. Unfortunately, high-dose fulvestrant was not approved in Japan until the end of 2011, so we could not include it in the present study. Our group of investigators is conducting another comparative study of high-dose fulvestrant with TOR120 in patients with AI-unresponsive tumors.

The three preceding options all target ER signaling, but some breast cancers become resistant to such therapies. Several molecular mechanisms have been proposed to be responsible for endocrine resistance. Loss of ER expression, altered activity of ER coregulators, deregulation of apoptosis and cell cycle signaling, and hyperactive receptor tyrosine kinase (RTK) and stress/cell kinase pathways can collectively orchestrate the development and sustenance of pharmacologic resistance to endocrine therapy [15].

Thus, a fourth category of therapies involves membrane-bound receptors for growth factors, such as the human EGF receptor (HER) family or insulin like growth factor receptor, which are active even in estrogen-dependent tumors. Treatment that combines endocrine therapy with inhibition of these growth factor receptors, or molecularly targeted treatment to inhibit their signal transmission, can be effective. mTOR (mammalian target of rapamycin) is a serine/threonine kinase in the downstream Akt pathway, which strongly affects cell survival and proliferation.

Recently, the phase III, Borelo2 trial, found that combination treatment with everolimus, an mTOR inhibitor, and exemestane had a statistically significant beneficial effect compared with exemestane alone in ORR (7.4% vs. 0.4%, respectively), and PFS (10.6 months vs. 4.1 months, respectively) [16]. Furthermore, another randomized phase II trial, the TAMRAD trial, comparing the combination everolimus and tamoxifen with tamoxifen alone showed a better CBR (61% vs. 42%) and longer TTP (8.6 months vs. 4.5 months) for the combination [17]. Interestingly, the efficacy of their control arms was similar to our results. ORR of EXE in Bolero 2 trial was 0.4% and 2.2% in ours, and CBR of TAM was 42% in the TAM-RAD trial and 41.3% in ours (Table 4). This further increases our confidence in our results.

## Conclusions

In summary, our study suggests that TOR120 should be regarded favorably as a subsequent endocrine therapy for recurrent breast cancer with non-steroidal AI failure, though with due attention to adverse symptoms, such as nausea and general fatigue. When choosing a subsequent endocrine therapy, it is important to select one that has endocrine therapy which has different mechanisms from prior therapy.

## Competing interests

All authors have declared no conflicts of interest.

## Authors’ contributions

All authors have made substantial contributions to conception and design, or acquisition of data, or analysis and interpretation of data. All authors have been involved in drafting the manuscript or revising it critically for important intellectual content. All authors have given final approval of the version to be published.

## Pre-publication history

The pre-publication history for this paper can be accessed here:

http://www.biomedcentral.com/1471-2407/13/239/prepub
